# Reduced diagnostic accuracy of screening questionnaires in older psychiatric patients at risk for obstructive sleep apnea: an observational cohort study

**DOI:** 10.1007/s11325-026-03847-0

**Published:** 2026-11-06

**Authors:** Maximilian Bailer, Eva M. Stein, Maximilian I. Sprügel, Stefan Mestermann, Franziska Sonnauer, Julian A. Schwarz, Janine Utz, Philipp Spitzer, Johannes Kornhuber

**Affiliations:** 1 Department of Psychiatry and Psychotherapy, University Hospital Erlangen, Friedrich-Alexander-Universität Erlangen-Nürnberg, 91054 Germany Schwabachanlage 6, Erlangen, Germany; 2 Department of Neurology, University Hospital Erlangen, Friedrich- Alexander-Universität Erlangen-Nürnberg, Erlangen, Germany; 3 Department of Child and Adolescent Mental Health, University Hospital Erlangen, Friedrich-Alexander-Universität Erlangen-Nürnberg, Erlangen, Germany

**Keywords:** Obstructive sleep apnea, Sleep-disordered breathing, OSA screening, Screening questionnaires, Psychiatric patients, Older patients, Diagnostic accuracy, STOP-Bang

## Abstract

**Background:**

Obstructive sleep apnea (OSA) is highly prevalent among geriatric patients and is associated with increased mortality, reduced quality of life, depression, and cognitive decline. Early identification and treatment are particularly important in older psychiatric patients, in whom typical OSA symptoms overlap with psychiatric and aging-related symptoms. Screening questionnaires are used to identify patients at risk for OSA, but their performance in older psychiatric populations is largely unknown. This study compared the diagnostic accuracy of screening questionnaires between younger and older psychiatric inpatients at risk for OSA and assessed the discriminative value of chronological age alone.

**Patients and methods:**

Observational study of consecutive admissions to a psychiatric ward at University Hospital Erlangen (June 2016–December 2022). 128 screened patients at risk for OSA with valid overnight polygraphy and questionnaire data were analyzed (72 aged 18–64 years; 56 aged ≥ 65 years). Risk scores were calculated for STOP-Bang, STOP-B28, BOAH, GOAL, NoSAS and No-Apnea questionnaires. The reference standard was apnea-hypopnea-index (AHI) measured by polygraphy. Primary outcome measures were the area under the receiver operating characteristic curve (AUC), sensitivity and specificity for any OSA (AHI ≥ 5 events/h). The discriminative value of age and individual questionnaire components was evaluated in the entire cohort.

**Results:**

In the screened cohort of psychiatric inpatients, older patients had higher median AHI (7.7 vs. 1.8 events/h; *p* = 0.007), greater prevalence of any OSA (AHI ≥ 5: 73.2% vs. 47.2%; *p* = 0.003), deeper desaturations and longer apneas. In younger patients, questionnaire AUCs ranged 0.61–0.78. In the older subgroup, AUCs were 0.12–0.36 lower; BOAH had the highest AUC (0.59), while the other questionnaires showed AUCs of 0.38–0.49. The lower discrimination was mainly reflected in lower specificity. Chronological age yielded an AUC of 0.70 (95% CI, 0.61–0.80), whereas BMI, neck circumference, and symptom-based items showed poor discrimination.

**Conclusions:**

In our sample of psychiatric inpatients at risk for OSA, commonly used screening questionnaires demonstrated markedly reduced diagnostic performance in patients aged ≥ 65 years, largely reflecting lower specificity. Chronological age demonstrated moderate discriminative value. Further research is warranted regarding the accuracy of OSA screening in older patients, and a lower threshold for device-based diagnostics may warrant consideration.

**Supplementary Information:**

The online version contains supplementary material available at https://doi.org/10.1007/s11325-026-03847-0.

## Introduction

Obstructive sleep apnea (OSA) is a very prevalent condition, particularly among psychiatric patients [1–4], where it poses serious health risks such as increased mortality, reduced quality of life, and heightened susceptibility to depression [5–7]. Early identification and management of OSA are critical in psychiatric populations due to its potential to exacerbate the course of psychiatric disorders, such as depression [8, 9].

OSA is typically diagnosed using a sequential algorithm, starting with a structured clinical history and examination, followed by assessment with a polygraphy device, also referred to as a home sleep apnea test (HSAT), and confirmation of diagnosis and treatment initiation with in-lab polysomnography (PSG) [10–12]. As instrument-based diagnostics are resource-intensive and may not be readily available in all clinical settings, tools for structured clinical histories and examinations are of great importance. Consequently, screening questionnaires, such as the STOP-Bang, BOAH, GOAL, NoSAS and No-Apnea scores, have been developed. These questionnaires identify patients at high risk for OSA based on self-report of symptoms (e.g., snoring, daytime sleepiness, and observed apneas) and common risk factors (e.g., body mass index (BMI), age, gender, and hypertension) [13–20].

However, applying these tools to specific subgroups, such as psychiatric patients, introduces challenges. OSA symptoms like fatigue, mood disturbances, and cognitive impairment frequently overlap with psychiatric symptoms [21–23], complicating the clinical screening process. As symptom interpretation is affected by comorbidities, this may reduce the reliability of screening tools in psychiatric populations [24]. To date, most screening tools have not been specifically validated in psychiatric patients. To address this issue, we assessed the diagnostic accuracy of OSA screening questionnaires in a cohort of psychiatric patients in a previous study, revealing high sensitivity, but low specificity [25].

Older patients face additional challenges in the assessment of sleep apnea: According to the current International Classification of Sleep Disorders (ICSD-3-TR), OSA in adults is diagnosed at an Apnea-Hypopnea Index (AHI) of ≥ 15 events per hour, or at an AHI of ≥ 5 events per hour when accompanied by typical nocturnal observations, such as snoring or witnessed apneas, or by symptoms leading to impaired sleep-related quality of life, such as sleepiness, fatigue, or insomnia [26]. In contrast to the previous version, comorbidities alone are no longer sufficient to establish the diagnosis, which places greater weight on the correct attribution of symptoms. In older patients, however, potential OSA symptoms such as fatigue and sleepiness overlap with typical signs of aging, and older OSA patients may exhibit atypical or less pronounced OSA symptoms [27]. Moreover, conditions frequently associated with OSA, such as hypertension, coronary artery disease, stroke, heart failure, atrial fibrillation, type 2 diabetes mellitus, mood disorders, or cognitive impairment [28–30], are significantly more prevalent in older adults, even in the absence of OSA [31–33], which may further complicate symptom attribution and reduce the discriminative value of comorbidity-based questionnaire items. Consequently, the specificity of screening tools is lower in older patients [34]. To address this, adaptions of the current standard screening questionnaires for older patients have been proposed, including the STOP-B28 questionnaire, an adaption of the STOP-Bang questionnaire [35]. However, there is limited evidence regarding the validity of screening questionnaires in the subgroup of older psychiatric patients, where diagnosing and treating OSA is critically important due to the high prevalence and significant impact on both physical and mental health.

This study aims to address this gap by comparing the diagnostic accuracy of the STOP-Bang, STOP-B28, BOAH, GOAL, NoSAS, and No-Apnea questionnaires between younger (aged < 65 years) and older (aged ≥ 65 years) psychiatric inpatients. We hypothesize that age-related physiological changes, the overlap of OSA symptoms with aging and psychiatric symptoms, and differing comorbidity profiles impair questionnaire performance in the older group, which would limit the clinical utility of questionnaire-based screening and support the need for age-adapted screening strategies.

## Methods

### Study subjects and study design

This observational cohort study is part of the UKER-OSA-PS study (Universitätsklinikum Erlangen study of obstructive sleep apnea in psychiatric patients), previously described in detail [25, 36]. The study included consecutive patients admitted to the private psychiatric ward at University Hospital Erlangen, Germany, between June 1, 2016, and December 31, 2022.

### Eligibility criteria

Inclusion criteria were adult patients (≥ 18 years) admitted with a psychiatric diagnosis confirmed by ICD-10 criteria [​Organic and Neurodegenerative Disorders (F00.x; F01.x; F02.x; F03; F04; F05.x; F06.x; F07); Substance Use Disorders (F10.x-F19.x), Psychotic Disorders (F20.x; F21; F22.x; F23.x; F24; F25.x); Affective Disorders (F30.x; F31.x; F32.x; F33.x; F34.x); Unipolar Depressive Disorder (F32.​x; F33.x); Bipolar Disorder (F30.x; F31.x); Neurotic, Stress-related and Somatoform Disorders (F40.x-​F48.x)] [37] and capable of completing the STOP-Bang questionnaire and undergoing polygraphy. Exclusion criteria included a prior OSA diagnosis, incomplete data for questionnaire calculations, or insufficient polygraphy recording time (< 4 h for flow or pulse signals).

### Age-based grouping

To assess the effect of age on the diagnostic accuracy of OSA screening tools, patients were stratified into “older” (≥ 65 years) and “younger” (< 65 years) patients in accordance with the German Society for Geriatrics guidelines, which define older adults as those aged 65 years or older [38].

### Procedures

#### Diagnostic algorithm

At admission all patients completed the STOP-Bang questionnaire. Per local protocol, overnight polygraphy was recommended for patients with STOP-Bang ≥ 3 or when clinicians documented a clinical suspicion of OSA; polygraphy was therefore not applied uniformly to all admissions. Overnight polygraphy was performed with the Somnocheck micro CARDIO^®^ home sleep apnea testing device (Weinmann GmbH, Hamburg, Germany), which has been validated against PSG in psychiatric inpatients, as previously described [25, 36]. Polygraphy/HSAT is an accepted diagnostic modality for OSA per current AASM guidelines [39]. In addition, the device derives an autonomic arousal index (AAI) from the photoplethysmographic pulse wave signal, defined as a pulse rate increase ≥ 20%, a pulse wave amplitude attenuation ≥ 40%, or a pulse wave amplitude attenuation ≥ 35% combined with a pulse rate increase ≥ 15% relative to baseline [40].

#### Questionnaires

All eligible patients completed the STOP-Bang questionnaire at the time of admission. All data required to calculate the STOP-B28, BOAH, GOAL, NoSAS and No-Apnea scores were available. A detailed description and analysis of the questionnaires can be found in a previous publication [25] and in Supplementary Table 1.

##### STOP-Bang and STOP-B28 questionnaires

The STOP-Bang questionnaire includes 8 questions regarding snoring, tiredness, observed apneas,​ high blood pressure, body mass index (BMI > 35 kg/m²), age (> 50 years), neck circumference (> 41 cm for females, > 43 cm for males), and male gender. A total score of ≥ 5 indicates a high risk, 3–4 indicates intermediate risk, and ≤ 2 indicates low risk [14, 15]. The STOP-B28 questionnaire, a modified version of the STOP-Bang score developed specifically for older patients, excludes the items age, neck circumference and gender, and uses a lower cut-off for obesity (BMI > 28 kg/m^2^) [41]. A STOP-B28 score of 0 or 1 is considered low risk for OSA.

##### BOAH questionnaire

The BOAH score assesses OSA risk using four variables: BMI, observed apneas, age, and hypertension. Points are assigned as follows: BMI ≥ 30 kg/m² (1 point) or ≥ 35 kg/m² (2 points); self-reported observed apneas (1 point); age ≥ 50 years (1 point); and arterial hypertension (1 point). Elevated risk of OSA is indicated by a total score of 2 or more points [16, 42].

##### GOAL questionnaire

The GOAL questionnaire uses four parameters: male gender (1 point), obesity (BMI ≥ 30 kg/m², 1 point), age ≥ 50 years (1 point), and loud snoring (1 point). A high risk of OSA is indicated by a score of 2 or more points [17, 18].

##### NoSAS questionnaire

The NoSAS score combines five variables: neck circumference (> 40 cm, 4 points), obesity (BMI 25–29.9 kg/m², 3 points; BMI ≥ 30 kg/m², 5 points), snoring (2 points), age (> 55 years, 4 points), and male gender (2 points). A high risk of OSA is indicated by a total score of 8 or more points [19].

##### No-Apnea questionnaire

The No-Apnea score evaluates risk based solely on neck circumference and age. Points are assigned as follows: neck circumference 37.0–39.9 cm (1 point), 40.0–42.9 cm (3 points), or ≥ 43.0 cm (6 points); and age 35–44 years (1 point), 45–54 years (2 points), or ≥ 55 years (3 points). A score of ≥ 3 indicates a high risk of OSA [20].

### Analysis

#### Data acquisition

Demographics, comorbidities, and in-hospital metrics were extracted from digitalized medical records using standardized protocols. In addition to general clinical data (age, sex, body-mass index) and medical comorbidities (e.g., hypertension, diabetes, cardiovascular disease), we recorded psychiatric characteristics including principal psychiatric diagnosis, current psychotropic medication, and Beck Depression Inventory-II (BDI-II) score at admission. The primary outcome was the diagnostic accuracy of each questionnaire, measured by sensitivity, specificity, and area under the ROC curve (AUC) at AHI ≥ 5 events/hour. This study was conducted according to the Strengthening the Reporting of Observational Studies in Epidemiology (STROBE) guidelines [43].

#### Statistical analysis

Statistical analyses were performed using IBM SPSS Statistics, version 29.0, and Microsoft Excel, version 2019. Categorical variables are reported as counts (percentages) and were compared with the χ² test or Fisher’s exact test, as appropriate. Continuous variables are presented as medians [interquartile ranges (IQRs)] and were compared between groups using the Mann-Whitney U test. All tests were two-sided and *p* < 0.05 was considered statistically significant. To assess diagnostic accuracy, the area under the receiver operating characteristic (ROC) curve was analyzed and sensitivity (proportion of true positives among all individuals with the condition; true positive / [true positive + false negative]) and specificity (proportion of true negatives among all individuals without the condition; true negative / [true negative + false positive]) were calculated through cross-tabulation. 95% confidence intervals for sensitivity and specificity were calculated using the Wilson score method. To explore the discriminative value of individual predictors in the entire cohort, ROC curves were calculated for the continuous variables age, BMI, and neck circumference, and sensitivity and specificity were calculated for the binary items snoring, daytime tiredness, observed apneas, hypertension, and male sex.

## Results

### Study population and baseline characteristics

**Fig. 1:**
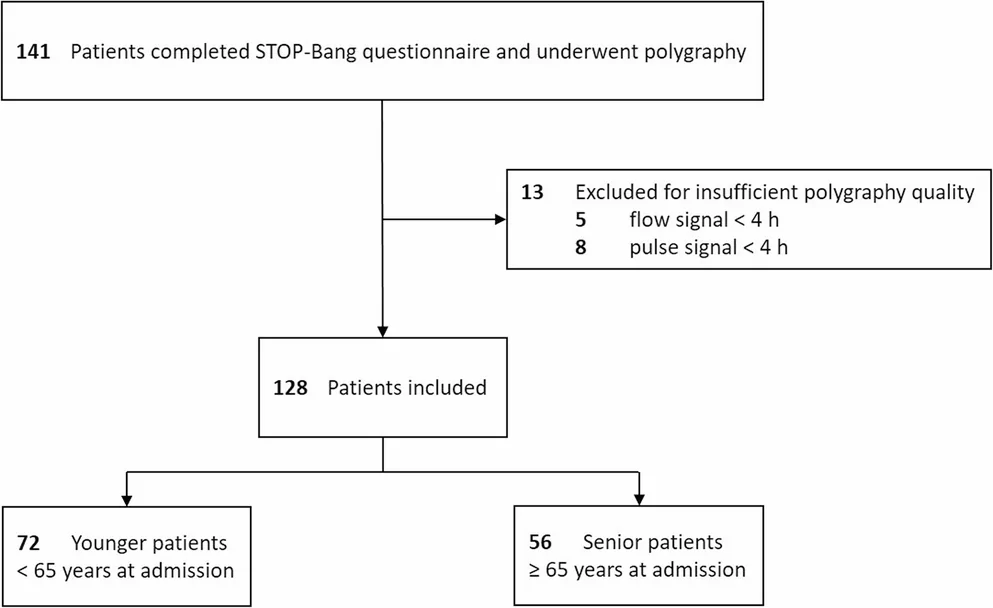
Flow chart of study participants

A total of 141 patients were included in this study (Fig. 1). Thirteen patients were excluded due to insufficient polygraphy quality. Overall, there were 128 patients included in the final analysis: 72 younger patients aged 18–64 years and 56 older patients aged ≥ 65 years (Table 1). The median age was 53.5 years [IQR, 40.0–59.0] for younger patients and 75.0 years [IQR, 71.0–77.8] for older patients (*p* < 0.001). Female patients made up 38.9% [28/72] of the younger group and 41.1% [23/56] of the older group (*p* = 0.8). Neither the median duration of hospital stay (34.5 days [IQR, 17.3–49.0] vs. 28.5 days [IQR, 3.5–43.8]; *p* = 0.2) nor the median interval between admission and polygraphy (2.0 days [IQR, 0.0–4.8] vs. 1.0 day [IQR, 0.0–5.0]; *p* = 0.6) differed significantly between younger and older patients.

**Table 1:** Baseline demographics and clinical characteristics

Variable	Younger Patients Age: 18–64 (*n* = 72)	Older Patients Age ≥ ​65 (*n* = 56)	*p*
Demographics			
Sex			
Female, No. (%)	​28 (38.9)	23 (41.1)	0.8
Male, No. (%)	44 (61.1)	33 (58.9)	0.8
Age, median (IQR), y	53.5 (40.0–59.0)	75.0 (71.0–77.8)	**< 0.001**
Adiposity			
BMI, median (IQR), kg/m2	27.6 (23.6–31.3)	26.1 (23.0–28.1)	**0.03**
BMI 30–34.9 kg/m^2^, No. (%)	13 (18.1)	5 (8.9)	0.1
BMI 35–39.9 kg/m^2^, No. (%)	6 (8.3)	3 (5.4)	0.7
BMI ≥ 40 kg/m^2^, No. (%)	4 (5.6)	0 (0.0)	0.1
BDI-II score at Admission, median (IQR) ^a^	27.0 (17.5–37.0)	20.0 (8.0–26.8)	**0.003**
Duration of Stay, median (IQR), d	34.5 (17.3–49.0)	28.5 (3.5–43.8)	0.2
Interval Admission to Polygraphy, median (IQR), d	2.0 (0.0–4.8)	1.0 (0.0–5.0)	0.6
Private Insurance, No. (%)	62 (86.1)	52 (92.9)	0.2
Psychiatric Diagnoses			
Organic and Neurodegenerative Disorders, No. (%)	13 (18.1)	20 (35.7)	**0.02**
Substance Use Disorders, No. (%)	15 (20.8)	13 (23.2)	0.8
Psychotic Disorders, No. (%)	2 (2.8)	2 (3.6)	0.8
Affective Disorders, No. (%)	59 (81.9)	41 (73.2)	0.2
Unipolar Depressive Disorder, No. (%)	55 (76.4)	38 (67.9)	0.3
Bipolar Disorder, No. (%)	4 (5.6)	3 (5.4)	> 0.95
Neurotic/Stress-related/Somatoform disorders, No. (%)	26 (36.1)	18 (32.1)	0.6
Non-psychiatric Diagnoses			
Pulmonary Diseases, No. (%)	6 (8.3)	6 (10.7)	0.7
COPD, No. (%)	1 (1.4)	2 (3.6)	0.6
Asthma, No. (%)	5 (6.9)	2 (3.6)	0.5
Cardiovascular Diseases, No. (%)	32 (44.4)	46 (82.1)	**< 0.001**
Arterial Hypertension, No. (%)	30 (41.7)	43 (76.8)	**< 0.001**
Atrial Fibrillation, No. (%)	2 (2.8)	9 (16.1)	**0.01**
Kidney Disorders, No. (%)	5 (6.9)	9 (16.1)	0.1
Hypothyroidism, No. (%)	15 (20.8)	10 (17.9)	0.​7
Substance Use History, No. (%) ^b^	44 (61.1)	23 (41.1)	**0.02**
Active Nicotine Use, No. (%) ^c^	25 (34.7)	9 (16.1)	**0.02**
Former Smoker, No. (%) ^d^	8 (11.1)	8 (14.3)	0.6
Regular Alcohol Consumption, No. (%) ^e^	20 (27.8)	12 (21.4)	0.4
Cannabis or Illicit Substance Use, No. (%) ^f^	13 (18.1)	3 (5.4)	**0.03**

a. Data available for 57 of 72 younger patients and 46 of 56 older patients

b. Substance Use History was defined as a self-reported prior use of nicotine, alcohol, cannabis or illicit substances

c. Active Nicotine Use was defined as self-reported current use of nicotine products or abstinence shorter than six months

d. Former smokers were defined as individuals with a self-reported history of smoking who had abstained for more than six months

e. Regular alcohol consumption was defined as self-reported drinking on at least three days per week

f. Cannabis or illicit substance use was defined as any self-reported use of cannabis or other illegal drugs at admission

Continuous and ordinal variables are reported as median (IQR); categorical variables as n (%). P values were obtained using Mann–Whitney U test for continuous variables and χ² test or Fisher’s exact test for categorical variables, as appropriate. *BMI  *body mass index, *No*. number, *BDI-II*  Beck Depression Inventory-II, *COPD  *chronic obstructive pulmonary disease

The median BMI was significantly higher in younger patients (27.6 kg/m² [IQR, 23.6–31.3]) compared to older patients (26.1 kg/m² [IQR, 23.0–28.1]; *p* = 0.03). Grade 1 obesity (BMI 30–34.9 kg/m²) was present in 18.1% [13/72] of younger patients and 8.9% [5/56] of older patients (*p* = 0.1), while grade 2 obesity (BMI 35–39.9 kg/m²) was found in 8.3% [6/72] and 5.4% [3/56], respectively (*p* = 0.7).

Among psychiatric diagnoses, organic and neurodegenerative disorders were significantly more common in older patients (35.7% [20/56]) than in younger patients (18.1% [13/72]; *p* = 0.02). Affective disorders were the most common psychiatric diagnoses in both groups, affecting 81.9% [59/72] of younger patients and 73.2% [41/56] of older patients (*p* = 0.2). Among these, unipolar depressive disorder was the predominant subtype, observed in 76.4% [55/72] of younger patients and 67.9% [38/56] of older patients (*p* = 0.3). The BDI-II score at admission was significantly higher in younger patients (27.0 [IQR, 17.5–37.0]) than in older patients (20.0 [IQR, 8.0–26.8]; *p* = 0.003). In addition, we documented the use of major psychiatric medication classes (antidepressants, antipsychotics, lithium, sedatives, anticonvulsants, and antidementia drugs) at the time of admission. No significant differences in medication use were observed between age groups (Supplementary Table 2).

Cardiovascular diseases were significantly more common in older patients, affecting 82.1% [46/56] compared to 44.4% [32/72] of younger patients (*p* < 0.001). Among these, arterial hypertension was observed in 76.8% [43/56] of older patients and 41.7% [30/72] of younger patients (*p* < 0.001). Additionally, atrial fibrillation occurred more frequently in older patients (16.1% [9/56]) compared to younger patients (2.8% [2/72]; *p* = 0.01).

Substance use history was more common in younger patients (61.1% [44/72] vs. 41.1% [23/56]; *p* = 0.02), particularly illicit drug or cannabinoid use, which was reported in 18.1% [13/72] of younger patients and 5.4% [3/56] of older patients (*p* = 0.03), as well as active nicotine use, observed in 34.7% [25/72] of younger patients compared to 16.1% [9/56] of older patients (*p* = 0.02).

### OSA questionnaire and polygraphy results

Screening questionnaire scores did not differ significantly between younger and older patients for STOP-Bang (3.0 [IQR, 3.0–5.0] vs. 4.0 [IQR, 3.0–5.0]; *p* = 0.3), STOP-B28 (3.0 [IQR, 2.0–3.0] vs. 2.0 [IQR, 2.0–3.0]; *p* = 0.2), GOAL (3.0 [IQR, 2.0–3.0] vs. 2.0 [IQR, 2.0–3.0]; *p* = 0.6), and No-Apnea (4.5 [IQR, 3.0–8.0] vs. 4.0 [IQR, 3.0–6.0]; *p* = 0.4) (Table 2). Due to the sequential screening algorithm, where polygraphy was performed in patients with elevated OSA risk according to clinical screening including the STOP-Bang score, there was an elevated score range in both subgroups. There were significant differences in the BOAH and NoSAS questionnaire scores between the two age groups: The median BOAH score was 2.0 [IQR, 1.0–3.0] in younger patients compared to 2.0 [IQR, 2.0–3.0] in older patients (*p* = 0.04), reflecting an elevated and more concentrated score range among older patients. The NoSAS score was significantly lower in older patients (5.0 [IQR, 3.0–11.0]) compared to younger patients (10.0 [IQR, 4.0–11.0]; *p* = 0.02).

**Table 2:** Questionnaire scores and overnight polygraphic examination data

Variable	Younger Patients Age: 18–64 (*n* = 72)	Older Patients Age ≥ 65 (*n* = 56)	*p*
Questionnaire Data			
STOP–Bang score, median (IQR)	3.0 (3.0–5.0)	4.0 (3.0–5.0)	0.3
STOP–B28 score, median (IQR)	3.0 (2.0–3.0)	2.0 (2.0–3.0)	0.2
BOAH score, median (IQR)	2.0 (1.0–3.0)	2.0 (2.0–3.0)	**0.04**
GOAL score, median (IQR)	3.0 (2.0–3.0)	2.0 (2.0–3.0)	0.6
NoSAS score, median (IQR)	10.0 (4.0–11.0)	5.0 (3.0–11.0)	**0.02**
No–Apnea score, median (IQR)	4.5 (3.0–8.0)	4.0 (3.0–6.0)	0.4
Polygraphic Examination Data			
Apneas and Hypopneas			
Apnea–Hypopnea–Index (AHI), median (IQR), events/h	1.8 (0.5–5.6)	7.7 (4.8–15.5)	**0.007**
Any OSA, No. (%) ^a^	34 (47.2)	41 (73.2)	**0.003**
Moderate to Severe OSA, No. (%) ^a^	8 (11.1)	16 (28.6)	**0.01**
Severe OSA, No. (%) ^a^	2 (2.8)	4 (7.1)	0.4
Apnea–Index (AI), median (IQR), events/h^b^	1.3 (0.5–4.0)	4.1 (2.0–11.8)	**< 0.001**
Hypopnea–Index (HI), median (IQR), events/h^b^	2.8 (1.3–4.7)	3.1 (1.8–5.7)	0.2
Longest Apnea, median (IQR), s^b^	19.5 (15.0–32.5)	31.0 (23.0–46.0)	**< 0.001**
Mean Apnea duration, median (IQR), s^b^	14.0 (12.0–16.0)	16.0 (15.0–19.0)	**< 0.001**
Snoring, median (IQR), % of measured time^b^	5.5 (0.0–20.5)	5.0 (1.0–15.5)	0.9
Oxygen Saturation			
Oxygen–Desaturation–Index (ODI), median (IQR), events/h	1.6 (0.5–4.0)	4.1 (1.2–8.2)	**0.007**
Duration below 95% SpO2, median (IQR), % of measured time	11.0 (2.0–53.5)	38.5 (17.3–87.8)	**< 0.001**
Min. SpO_2_, median (IQR), %	85.0 (80.0–89.0)	83.0 (79.0–85.8)	**0.05**
Average SpO_2_, median (IQR), %	95.0 (94.0–96.0)	94.0 (92.3–95.0)	**< 0.001**
Pulse Oximetry Data			
Average Pulse, median (IQR), bpm	65.0 (57.0–69.8)	63.5 (58.0–70.0)	0.8
Min. Pulse, median (IQR), bpm	44.0 (39.3–48.8)	46.0 (40.3–50.0)	0.2
Max. Pulse, median (IQR), bpm	96.0 (89.3–105.0)	93.0 (87.0–98.8)	**0.03**

a. Any OSA: AHI ≥ 5; Moderate to Severe OSA: AHI ≥ 15; Severe OSA: AHI ≥ 30

b. Data available for 60 of 72 younger patients and 49 of 56 older patients

Continuous variables are shown as median (IQR); categorical variables as n (%). P values were calculated using Mann–Whitney U test, χ² test, or Fisher’s exact test, as appropriate. SpO₂ = peripheral oxygen saturation

Obstructive sleep apnea (OSA) was significantly more prevalent in older patients compared to younger patients: The median AHI was significantly higher in older patients (7.7 events/h [IQR, 4.8–15.5]) than in younger patients (1.8 events/h [IQR, 0.5–5.6]; *p* = 0.007). Any OSA (AHI ≥ 5) was present in 73.2% [41/56] of older patients and 47.2% [34/72] of younger patients (*p* = 0.003). Moderate to severe OSA (AHI ≥ 15) occurred in 28.6% [16/56] of older patients and 11.1% [8/72] of younger patients (*p* = 0.01). The proportion of patients with severe OSA (AHI ≥ 30) was low in both groups, affecting 7.1% [4/56] of older patients and 2.8% [2/72] of younger patients (*p* = 0.4).

Apart from the higher prevalence, OSA severity was also greater in older patients: Both the mean apnea duration (16.0 s [IQR, 15.0–19.0]) and the longest apnea duration (31.0 s [IQR, 23.0–46.0]) were significantly longer in older patients compared to younger patients (14.0 s [IQR, 12.0–16.0] and 19.5 s [IQR, 15.0–32.5]; *p* < 0.001 for both). Additionally, older patients exhibited a higher desaturation index (4.1 events/h [IQR, 1.2–8.2] vs. 1.6 events/h [IQR, 0.5–4.0]; *p* = 0.007), longer duration below 95% SpO₂ (38.5% [IQR, 17.3–87.8] vs. 11.0% of measured time [IQR, 2.0–53.5]; *p* < 0.001), and a lower minimum SpO₂ (83.0% [IQR, 79.0–85.8] vs. 85.0% [IQR, 80.0–89.0]; *p* = 0.05). The autonomic arousal index (AAI), a pulse wave–derived marker of autonomic activation, was higher in younger patients (19.2 events/h [IQR, 12.8–27.7]) compared to older patients (7.3 events/h [IQR, 4.5–10.1]; *p* < 0.001). However, in data from our previous validation study of the device, the AAI was not correlated with the PSG arousal index (Supplementary Table 3).

### Receiver operating characteristic curve analysis

**Fig. 2:**
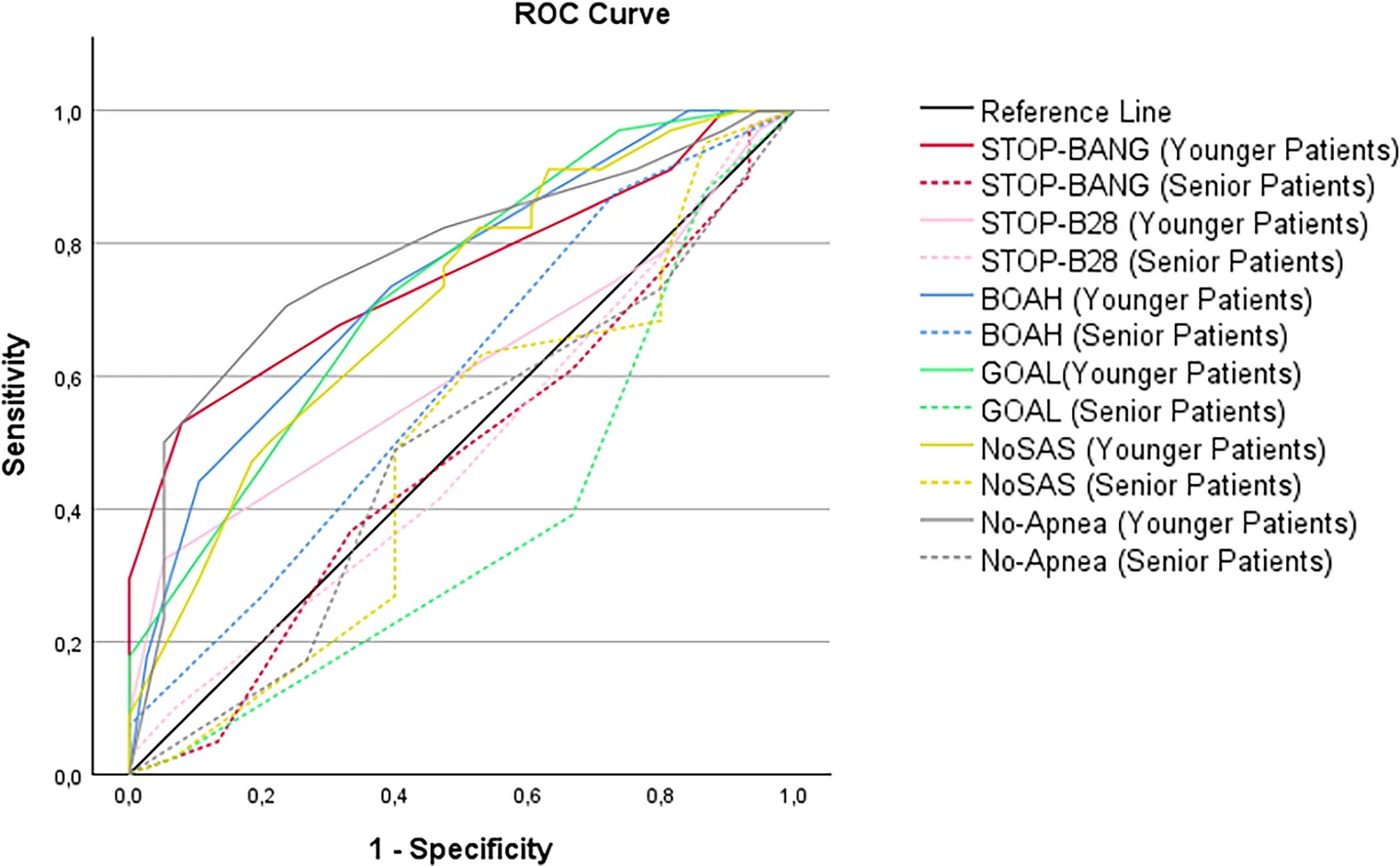
Receiver operating characteristic (ROC) curves of the questionnaires

Receiver Operating Characteristic (ROC) curves were used to compare the diagnostic accuracy of screening questionnaires for the diagnosis of any OSA (AHI ≥ 5 events/h) between younger and older patients (Fig. 2). In our screened cohort, the Area Under the Curve (AUC) values were lower in older patients for all questionnaires, with Δ AUCs ranging from 0.12 to 0.36 (Table 3). The No-Apnea score, which displayed the highest AUC in younger patients of 0.78 [95% CI, 0.67–0.89], showed a lower AUC of 0.48 [95% CI, 0.31–0.66; Δ AUC, 0.30; *p* = 0.005] in older patients. Significantly lower AUCs were also observed in older patients in the STOP-Bang (younger patients, AUC, 0.75 [95% CI, 0.64–0.87] vs. older patients, AUC, 0.48 [95% CI, 0.30–0.65]; Δ AUC, 0.28; *p* = 0.009) and GOAL questionnaires (younger patients, AUC, 0.73 [95% CI, 0.62–0.85] vs. older patients, AUC, 0.38 [95% CI, 0.20–0.55]; Δ AUC, 0.36; *p* = 0.001); and a similar trend was observed for NoSAS (younger patients, AUC, 0.71 [95% CI, 0.59–0.83] vs. older patients, AUC, 0.49 [95% CI, 0.30–0.67]; Δ AUC, 0.22; *p* = 0.05). The STOP-B28 score, despite being designed for older patients, did not demonstrate superior performance in this group compared to the other questionnaires and exhibited the lowest AUC in younger patients (younger patients, AUC, 0.61 [95% CI, 0.47–0.74] vs. older patients, AUC, 0.49 [95% CI, 0.32–0.66]; Δ AUC, 0.12; *p* = 0.3). The BOAH questionnaire had the highest accuracy in older patients among all the questionnaires (younger patients, AUC, 0.74 [95% CI, 0.63–0.86] vs. older patients, AUC, 0.59 [95% CI, 0.42–0.76]; Δ AUC, 0.15; *p* = 0.1). ROC analysis for moderate to severe OSA (AHI ≥ 15 events/h) revealed no significant differences between younger and older patients (Supplementary Fig. 1 and Supplementary Table 4). However, the reliability of this finding is limited by the low number of patients in the category of moderate to severe OSA (8 younger patients and 16 older patients).

**Table 3:** Comparison of the area under the receiver operating characteristic curves for any OSA (AHI ≥ 5)

Questionnaire	AUC (95% CI)	AUC (95% CI)	Δ AUC	*p*
	Younger Patients	Older Patients		
STOP-Bang	0.75 (0.64–0.87)	0.48 (0.30–0.65)	0.28	0.009
STOP-B28	0.61 (0.47–0.74)	0.49 (0.32–0.66)	0.12	0.3
BOAH	0.74 (0.63–0.86)	0.59 (0.42–0.76)	0.15	0.1
GOAL	0.73 (0.62–0.85)	0.38 (0.20–0.55)	0.36	0.001
NoSAS	0.71 (0.59–0.83)	0.49 (0.30–0.67)	0.22	0.05
No-Apnea	0.78 (0.67–0.89)	0.48 (0.31–0.66)	0.30	0.005

ΔAUC indicates difference between AUCs; *AHI  *apnea–hypopnea index, *AUC  *area under the curve

### Diagnostic performance of the questionnaires

Diagnostic performance of each questionnaire for detecting OSA (AHI ≥ 5 events/h) is presented in Table 4. Overall, accuracy was consistently higher in younger patients across most questionnaires. For the STOP‑Bang score at cut‑off ≥ 3, sensitivity was similar in both age cohorts (younger patients, 91.2% vs. older patients, 90.2%), but specificity was higher in younger patients (younger patients, 18.4% vs. older patients, 6.7%). Raising the cut-off to ≥ 4 led to a substantial increase in specificity among younger patients (younger patients, 68.4% vs. older patients, 33.3%), while sensitivity was comparable between the cohorts (younger patients, 67.6% vs. older patients, 61.0%). The STOP‑B28, designed for older adults, showed nearly identical performance at cut‑off ≥ 2 in both groups (younger patients: sensitivity, 79.4%, specificity, 18.4% vs. older patients: sensitivity, 78.0%, specificity, 20.0%), though its sensitivity was lower than that of the original STOP‑Bang in both groups. Among the remaining tools, only the BOAH score achieved higher sensitivity in older patients at its primary threshold of ≥ 2 (younger patients, 73.5% vs. older patients, 87.8%), but specificity was lower (younger patients, 60.5% vs. older patients, 26.7%). Both the GOAL (younger patients: sensitivity, 97.1%, specificity, 26.3% vs. older patients: sensitivity, 87.8%, specificity, 13.3%) and NoSAS (younger patients: sensitivity, 82.4%, specificity, 39.5% vs. older patients: sensitivity, 68.3%, specificity, 20.0%) questionnaires performed better in younger patients. By design, all older patients scored ≥ 3 on the No‑Apnea score; at this threshold its sensitivity in younger patients reached 91.2% with 23.7% specificity. Increasing the cut‑off to ≥ 4 balanced performance in younger patients (sensitivity 82.4%, specificity 52.6%), while it remained poor in older patients (sensitivity 73.2%, specificity 20.0%).

**Table 4:** Predictive performance of questionnaires for AHI ≥ 5

Questionnaire	Cut-off	Younger Patients		Older Patients	
		Sensitivity	Specificity	Sensitivity	Specificity
STOP-Bang	≥ 3	91.2 (77.0–97.0)	18.4 (9.2–33.4)	90.2 (77.5–96.1)	6.7 (1.2–29.8)
	≥ 4	67.6 (50.8–80.9)	68.4 (52.5–80.9)	61.0 (45.7–74.3)	33.3 (15.2–58.3)
	≥ 5	52.9 (36.7–68.5)	92.1 (79.2–97.3)	36.6 (23.6–51.9)	66.7 (41.7–84.8)
STOP-B28	≥ 2	79.4 (63.2–89.7)	18.4 (9.2–33.4)	78.0 (63.3–88.0)	20.0 (7.0–45.2)
	≥ 3	58.8 (42.2–73.6)	52.6 (37.3–67.5)	41.5 (27.8–56.6)	53.3 (30.1–75.2)
BOAH	≥ 2	73.5 (56.9–85.4)	60.5 (44.7–74.4)	87.8 (74.5–94.7)	26.7 (10.9–52.0)
	≥ 3	44.1 (28.9–60.5)	89.5 (75.9–95.8)	26.8 (15.7–41.9)	80.0 (54.8–93.0)
GOAL	≥ 2	97.1 (85.1–99.5)	26.3 (15.0–42.0)	87.8 (74.5–94.7)	13.3 (3.7–37.9)
	≥ 3	70.6 (53.8–83.2)	63.2 (47.3–76.6)	39.0 (25.7–54.3)	33.3 (15.2–58.3)
NoSAS	≥ 8	82.4 (66.5–91.7)	39.5 (25.6–55.3)	68.3 (53.0–80.4)	20.0 (7.0–45.2)
	≥ 9	82.4 (66.5–91.7)	47.4 (32.5–62.7)	63.4 (48.1–76.4)	46.7 (24.8–69.9)
	≥ 10	76.5 (60.0–87.6)	52.6 (37.3–67.5)	48.8 (34.3–63.5)	60.0 (35.7–80.2)
No-Apnea	≥ 3	91.2 (77.0–97.0)	23.7 (13.0–39.2)	100 (91.4–100)	0 (0–20.4)
	≥ 4	82.4 (66.5–91.7)	52.6 (37.3–67.5)	73.2 (58.1–84.3)	20.0 (7.0–45.2)
	≥ 5	73.5 (56.9–85.4)	71.1 (55.2–83.0)	48.8 (34.3–63.5)	60.0 (35.7–80.2)

Values are given as % (95% CI). Validity indices were calculated for OSA defined as AHI ≥ 5. 95% confidence intervals were calculated using the Wilson score method. *AHI  *apnea–hypopnea index, *CI  *confidence interval

### Age and other questionnaire components as predictors of OSA

**Fig. 3:**
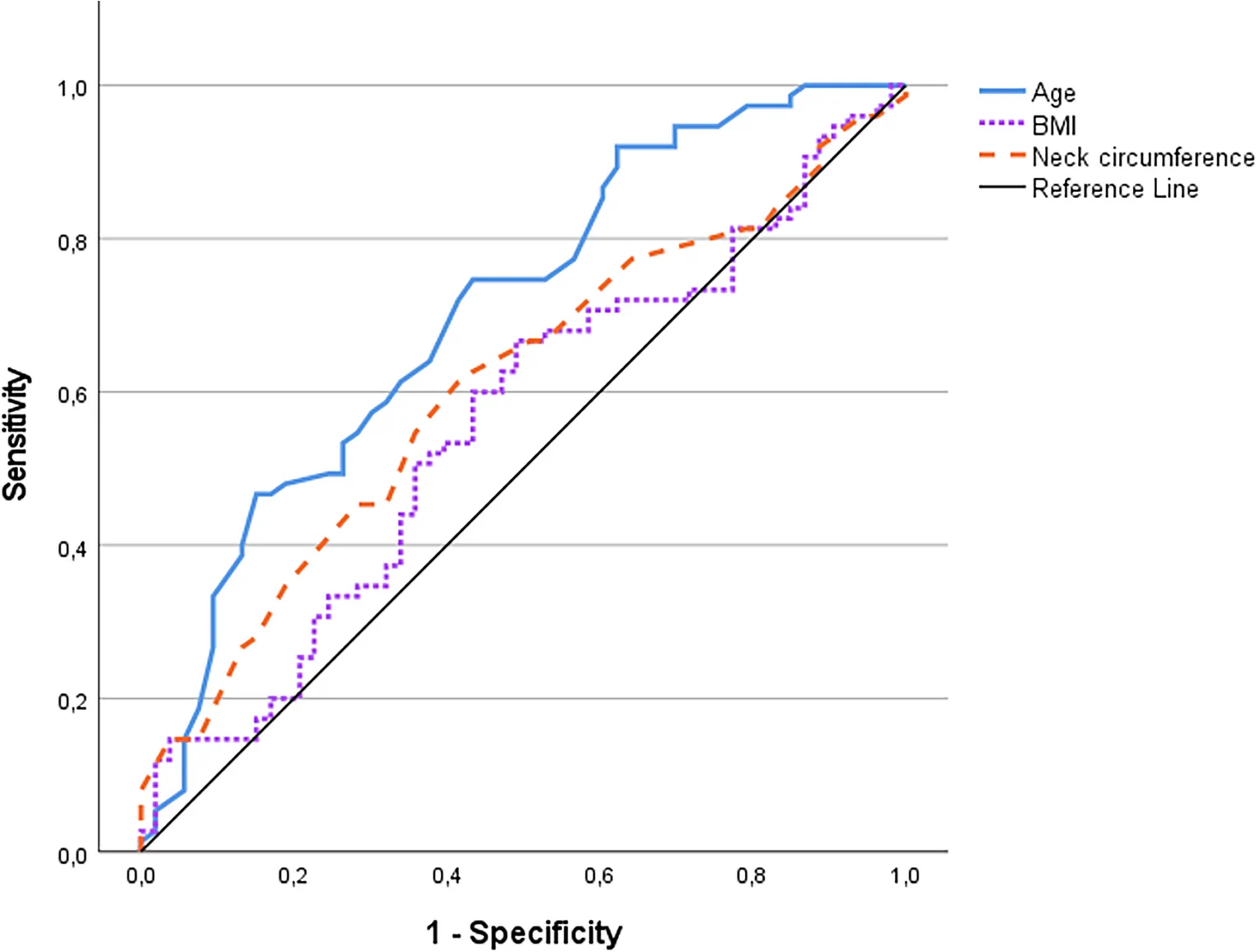
Receiver operating characteristic (ROC) curve of age, BMI, and neck circumference

ROC curve analysis was used to investigate age, BMI, and neck circumference as predictors of OSA (Fig. 3) in the entire cohort (*n* = 128). Here, age alone yielded an AUC of 0.70 [95% CI, 0.61–0.80] for the detection of any OSA (AHI ≥ 5 events/h), which exceeded the AUCs observed for several questionnaires in older patients. In contrast, neck circumference showed poor discrimination (AUC, 0.61 [95% CI, 0.51–0.71]; *p* = 0.03), and BMI did not discriminate significantly (AUC, 0.56 [95% CI, 0.46–0.66]; *p* = 0.3). Among binary items, the symptom-based items snoring, daytime tiredness, and observed apneas as well as male sex showed low discriminative value, with either low sensitivity or low specificity, whereas hypertension showed the most balanced profile (sensitivity, 69.3%; specificity, 54.7%) (Supplementary Table 5).

## Discussion

In this cohort of psychiatric inpatients at risk for OSA, obstructive sleep apnea (OSA) was both more prevalent and more severe in patients aged ≥ 65 years than in those < 65 years. Older patients showed higher median apnea–hypopnea indices, more pronounced desaturations, and longer apneic events. These observations are consistent with epidemiologic data identifying age as a strong risk factor for OSA [44–46], and underscore the clinical importance of early and accurate OSA detection in older psychiatric patients. Despite the greater objectively measured disease burden, median scores on the STOP-Bang, STOP-B28, BOAH, GOAL, NoSAS, and No-Apnea questionnaires were not higher in the older subgroup of psychiatric inpatients.

Receiver operating characteristic (ROC) analysis in the screened cohort showed greater discriminative accuracy of most questionnaires in younger than in older patients. In younger patients, AUCs for STOP-Bang, No-Apnea, NoSAS, and GOAL ranged from 0.71 to 0.78, modestly lower than the 0.86–0.94 (AHI ≥ 5) reported in sleep-clinic populations [17], but comparable to data from cohorts with major depressive disorder (0.73–0.77 for AHI ≥ 5) [47]. In older patients, absolute AUCs declined by 0.12 to 0.36 points. The BOAH questionnaire showed the highest AUC (0.59), whereas all other instruments performed poorly (AUCs 0.38–0.49, with confidence intervals including 0.5, indicating discrimination not better than chance). Because of the sequential diagnostic algorithm, absolute AUC estimates should be interpreted with caution; however, the relative decline in discrimination with advancing age in the verified cohort is notable and merits further evaluation.

The reduced discriminative performance of screening questionnaires in the subgroup of older patients appeared to be driven mainly by lower specificity rather than lower sensitivity: For instance, at its primary threshold of ≥ 2, the BOAH score showed higher sensitivity in older than in younger patients (87.8% vs. 73.5%), whereas specificity was lower (26.7% vs. 60.5%). Analogous age-related declines in specificity were evident for the STOP-Bang, GOAL, and No-Apnea instruments, consistent with prior reports in older cohorts [35]. Given the wide confidence intervals, particularly for specificity in older patients, these differences should be interpreted with caution.

There are few studies that have evaluated OSA screening questionnaire performance specifically in older adults, and none to our knowledge in a psychiatric population. In sleep-clinic cohorts the results are heterogeneous. In a Brazilian sleep clinic series that used a combined symptom + AHI definition of OSA, STOP-Bang retained robust discrimination in patients aged ≥ 60 years (AUC, 0.80; Sensitivity, 0.89; Specificity, 0.42) [48]. Another Brazilian sleep clinic study reported a STOP-Bang sensitivity of 0.71 and specificity of 0.41 for AHI ≥ 5 in older patients [49]. By contrast, Martins et al. found lower AUCs in an older cohort of community-dwelling adults aged ≥ 65 years: STOP-Bang and STOP-B28 yielded AUCs of around 0.64 for AHI ≥ 5 [35]. A validation study in patients with Parkinson disease reported AUCs of 0.66–0.76 for STOP-Bang, STOP-B28 and GOAL, but these estimates referred to AHI ≥ 15 and the mean age (≈ 64.5 years) was lower than that of our older subgroup, limiting direct comparison [41].

In summary, in our screened cohort of older psychiatric inpatients, the discriminative performance of screening questionnaires appeared lower than that reported in some non-psychiatric geriatric samples, though direct comparison is limited by differences in study design. While the sequential diagnostic algorithm with preferential testing of high-risk patients may have biased specificity and AUC downward in our sample, the relative deficit in older patients may also reflect factors specific to psychiatric inpatients. Symptom overlap may be a major contributor: fatigue, excessive daytime sleepiness, and cognitive dysfunction are core features of OSA but also occur in depression and other psychiatric disorders [23, 50]. This can inflate affirmative responses to symptom-based questionnaire items and increase false-positive rates, particularly in older patients. Consistent with this, symptom-based items such as snoring and daytime tiredness showed low discriminative value in our cohort. In older psychiatric patients this problem is compounded because OSA symptoms further overlap with common manifestations of aging, making correct attribution even more difficult [27]. Finally, the high prevalence of neurodegenerative disease in our cohort of older psychiatric inpatients may suggest cognitive heterogeneity that could influence questionnaire validity, though formal cognitive assessment was not available. Cognitive deficits may reduce the validity of self-reported histories through impaired recall, reduced insight, and response bias, eroding specificity and overall test discrimination [51, 52].

Psychotropic medication may have influenced both polygraphic findings and questionnaire responses. Benzodiazepines and Z-drugs can reduce upper airway muscle tone and arousal responses, and sedating antipsychotics and some antidepressants are associated with weight gain, all of which may aggravate sleep-disordered breathing [53, 54]. In addition, sedating medication may contribute to daytime tiredness and thereby to positive responses on symptom-based questionnaire items. In our sample, the use of major psychotropic medication classes at admission did not differ between younger and older patients, and polygraphy was performed early after admission (median interval, 1–2 days), so that medication at admission likely reflected medication during the recording night in most patients. Medication effects are therefore unlikely to account for the age-related differences in questionnaire performance observed in our study, although they may have influenced absolute estimates in both age groups. However, dosages, polypharmacy, and medication changes between admission and polygraphy could not be systematically assessed, and residual confounding by psychotropic medication cannot be excluded.

Another factor that may account for discrepant questionnaire performance is heterogeneity in the OSA reference definition. In line with previous validation studies of OSA screening questionnaires, OSA was defined by AHI thresholds, providing an objective reference standard that is independent of symptom reporting. According to the ICSD-3-TR, symptoms establishing the diagnosis of OSA at an AHI of 5–14 events/h include, among others, sleepiness, fatigue, insomnia, or other symptoms impairing sleep-related quality of life, as well as awakenings with breath holding, gasping, or choking [26]. Such symptoms are highly prevalent among psychiatric inpatients, particularly in those with depressive disorders [55]. We therefore consider AHI ≥ 5 to be of primary clinical relevance in this population, consistent with guideline recommendations to consider positive airway pressure therapy in symptomatic patients with mild OSA [56, 57].

Chronological age yielded an AUC of 0.70 in the ROC analysis of the entire cohort of psychiatric patients at risk for OSA. In contrast, BMI and neck circumference showed poor discrimination, and symptom-based items as well as male sex showed low discriminative value. This finding raises the hypothesis that advancing age may overshadow other conventional risk factors in determining OSA risk among psychiatric inpatients. Our data align with prior epidemiologic work showing that the effectiveness of conventional predictors, notably body-mass index, reported snoring and witnessed apneas, weakens with advancing age [44]. Moreover, male sex, a strong predictor in middle-aged cohorts, loses discriminatory value in older populations, in part because OSA prevalence in postmenopausal women rises and gender prevalence ratios converge with age [58–60]. As these analyses were performed in a cohort preselected for elevated OSA risk, they should be considered exploratory.

Differences in questionnaire design and performance have pragmatic implications. The STOP-B28 score showed low discriminatory accuracy in our sample both in younger (AUC, 0.61) and older (AUC, 0.49) patients. Paradoxically, NoSAS scores were lower among older patients in our sample despite greater OSA severity. This is potentially due to NoSAS’s substantial weighting of adiposity (BMI and neck circumference), which was less prevalent in the older subgroup of our screened cohort, and is consistent with the poor discriminative value of BMI and neck circumference in our cohort. By contrast, the BOAH questionnaire showed the highest AUC (0.59) and a high sensitivity (87.8%) in older patients, albeit with low specificity (26.7%). BOAH’s pragmatic advantages (no neck-circumference measurement required) may support its further evaluation as a quick screening tool in older patients.

In our screened cohort, validated questionnaires remained useful to triage younger psychiatric inpatients at risk for OSA. The No-Apnea questionnaire showed the highest AUC in younger patients, with high sensitivity at a cut-off of ≥ 3 and a more balanced performance at a cut-off of ≥ 4. In older patients in our screened cohort, the limited discrimination of all questionnaires suggests that reliance on questionnaires alone may be inadequate. Given the high prevalence [61], greater severity [62], and frequently atypical presentation of OSA in older patients [63], a lower threshold for direct device-based diagnostics (polygraphy/HSAT or in-lab PSG) may warrant consideration.

### Limitations and future directions

This study has several limitations. First, as polygraphy was performed preferentially in patients with STOP-Bang ≥ 3 or with clinical suspicion of OSA, our findings are subject to partial verification bias and spectrum bias and apply to psychiatric inpatients at risk for OSA rather than to all psychiatric admissions. Such sampling increases pre-test probability and may lead to an overestimation of sensitivity and an underestimation of specificity and AUC. However, reported prevalence estimates reach similarly high levels in geriatric samples (e.g., up to 75%) [58, 63] as in our screened cohort of older psychiatric patients at risk for OSA, and limited questionnaire discrimination has also been observed in such samples [35]. Second, the STOP-Bang questionnaire served both as part of the referral criterion and as an index test, which restricts the range of STOP-Bang scores and, to a lesser extent, of other questionnaires sharing items with it. STOP-Bang estimates should therefore be interpreted with particular caution. Third, this was a single-center study with a moderate sample size, particularly in the older subgroup, which limits the precision and generalizability of our estimates. Fourth, OSA was assessed by polygraphy rather than PSG; however, the device has been validated against PSG in this patient population [36], and polygraphy is an accepted diagnostic method for OSA [39].

Prospective studies are therefore warranted to confirm our findings. Specifically, future work should include larger, multicenter cohorts (including outpatient and community settings) with uniform application of device-based reference testing to obtain unbiased estimates of questionnaire accuracy. Studies should also formally quantify the impact of cognitive impairment and neurodegenerative disease on questionnaire reliability and assess whether recalibration or redevelopment of screening instruments improves discrimination in older psychiatric populations. Finally, research should evaluate pragmatic clinical pathways that prioritize early device-based testing for older psychiatric inpatients at elevated risk.

## Conclusions

In our cohort of psychiatric inpatients at risk for OSA, screening questionnaires retained moderate discriminative accuracy in younger patients but performed poorly in older patients, largely reflecting lower specificity. Chronological age alone showed moderate discriminative value. Our findings support considering a lower threshold for direct device-based testing in older psychiatric patients at risk for OSA, pending prospective validation.

## Supplementary Information

Below is the link to the electronic supplementary material.

Supplementary Material 1

## Data Availability

Datasets can be obtained from the corresponding author upon reasonable request.

## Abbreviations

AAI: Autonomic arousal index
AASM: American Academy of Sleep Medicine
AHI: Apnea-hypopnea index
AI: Apnea index
AUC: Area under the curve
BDI-II: Beck Depression Inventory-II
BMI: Body mass index
bpm: Beats per minute
CI: Confidence interval
COPD: Chronic obstructive pulmonary disease
d: Days
Δ: Change or difference, used here for Δ AUC
h: Hour
HI: Hypopnea index
HSAT: Home sleep apnea test
ICD-10: International Classification of Diseases, 10th Revision
ICSD-3-TR: International Classification of Sleep Disorders, Third Edition, Text Revision
IQR: Interquartile range
kg/m²: Kilograms per square meter
No: Number
ODI: Oxygen-desaturation index
OSA: Obstructive sleep apnea
PSG: Polysomnography
ROC: Receiver operating characteristic
SpO2: Peripheral oxygen saturation
STROBE: Strengthening the Reporting of Observational Studies in Epidemiology
UKER-OSA-PS: Universitätsklinikum Erlangen study of obstructive sleep apnea in psychiatric patients
y: Years

## Abbreviations

AAI: Autonomic arousal index
AASM: American Academy of Sleep Medicine
AHI: Apnea-hypopnea index
AI: Apnea index
AUC: Area under the curve
BDI-II: Beck Depression Inventory-II
BMI: Body mass index
bpm: Beats per minute
CI: Confidence interval
COPD: Chronic obstructive pulmonary disease
d: Days
Δ: Change or difference, used here for Δ AUC
h: Hour
HI: Hypopnea index
HSAT: Home sleep apnea test
ICD-10: International Classification of Diseases, 10th Revision
ICSD-3-TR: International Classification of Sleep Disorders, Third Edition, Text Revision
IQR: Interquartile range
kg/m²: Kilograms per square meter
No: Number
ODI: Oxygen-desaturation index
OSA: Obstructive sleep apnea
PSG: Polysomnography
ROC: Receiver operating characteristic
SpO2: Peripheral oxygen saturation
STROBE: Strengthening the Reporting of Observational Studies in Epidemiology
UKER-OSA-PS: Universitätsklinikum Erlangen study of obstructive sleep apnea in psychiatric patients
y: Years
