# Supplementary material for: Reduced diagnostic accuracy of screening questionnaires in older psychiatric patients at risk for obstructive sleep apnea: an observational cohort study
Source: Sleep Breath. 2026 Nov 6;30(6):306. doi: 10.1007/s11325-026-03847-0 (PMC13743650; doi:10.1007/s11325-026-03847-0)
Supplement: Supplementary file 1 [file 11325_2026_3847_MOESM1_ESM.docx]

Supplementary Material

# 1 Supplementary Figures and Tables

## 1.1 Supplementary Table 1. Summary of Screening Questionnaires

|  | **STOP-Bang** | | **STOP-B28** | | **BOAH** | | **GOAL** | | **NoSAS** | | **No-Apnea** | |
| --- | --- | --- | --- | --- | --- | --- | --- | --- | --- | --- | --- | --- |
|  |  | **Points** |  | **Points** |  | **Points** |  | **Points** |  | **Points** |  | **Points** |
| **Snoring** | Yes | 1 | Yes | 1 | - | 0 | Yes | 1 | Yes | 2 | - | 0 |
| **Tiredness** | Yes | 1 | Yes | 1 | - | 0 | - | 0 | - | 0 | - | 0 |
| **Observed Apnea** | Yes | 1 | Yes | 1 | Yes | 1 | - | 0 | - | 0 | - | 0 |
| **Hypertension** | Yes | 1 | Yes | 1 | Yes | 1 | - | 0 | - | 0 | - | 0 |
| **BMI (kg/m²)** | >35 | 1 | >28 | 1 | ≥30 | 1 | ≥30 | 1 | 25-29.9 | 3 | - | 0 |
|  |  |  |  |  | ≥35 | 2 |  |  | ≥ 30 | 5 |  |  |
| **Age (years)** | >50 | 1 | - | 0 | ≥50 | 1 | ≥50 | 1 | >55 | 4 | 35-44 | 1 |
|  |  |  |  |  |  |  |  |  |  |  | 45-54 | 2 |
|  |  |  |  |  |  |  |  |  |  |  | ≥55 | 3 |
| **Neck Circumference (cm)** | >41 (f); >43 (m) | 1 | - | 0 | - | 0 | - | 0 | >40 | 4 | 37.0-39.9 | 1 |
|  |  |  |  |  |  |  |  |  |  |  | 40.0-42.9 | 3 |
|  |  |  |  |  |  |  |  |  |  |  | ≥43.0 | 6 |
| **Gender** | Male | 1 | - | 0 | - | 0 | Male | 1 | Male | 2 | - | 0 |
| **Score Range** |  | 0-8 |  | 0-5 |  | 0-5 |  | 0-4 |  | 0-17 |  | 0-9 |
| **Cut-off** | ≥ 3: intermediate;  ≥ 5: high risk | | ≥ 2 | | ≥ 2 | | ≥ 2 | | ≥ 8 | | ≥ 3 | |

**Notes:**

BMI indicates body mass index; f, female; m, male.

## 1.2 Supplementary Table 2. Psychiatric Medication Use at Admission

| **Variable** | **Younger Patients** Age: 18-64  **(n=72)** | **Older Patients**  Age ≥ 65 **(n=56)** | **p** |
| --- | --- | --- | --- |
| Psychiatric Medication |  |  |  |
| Antidepressants, No. (%) | 44 (61.1) | 31 (55.4) | 0.5 |
| Antipsychotics, No. (%) | 17 (23.6) | 19 (33.9) | 0.2 |
| Lithium, No. (%) | 3 (4.2) | 0 (0) | 0.1 |
| Sedatives, No. (%) ^a^ | 17 (23.6) | 15 (26.8) | 0.7 |
| Anticonvulsants, No. (%) | 5 (6.9) | 3 (5.4) | 0.7 |
| Antidementia drugs, No. (%) | 3 (4.2) | 1 (1.8) | 0.4 |

**Notes:**

^a^. Sedatives were defined as patients taking zopiclone, zolpidem or benzodiazepines at admission.

Categorical variables are reported as n (%). P values were calculated using χ² test or Fisher's exact test, as appropriate. No significant differences in medication classes were observed between age groups.

## 1.3 Supplementary Table 3. Autonomic Arousal Index in Younger and Older Patients

| **Variable** | **Younger Patients** Age: 18-64  **(n=72)** | **Older Patients**  Age ≥ 65 **(n=56)** | **p** |
| --- | --- | --- | --- |
| AAI, median (IQR), events/h ^a^ | 19.2 (12.8-27.7) | 7.3 (4.5-10.1) | **< .001** |

**Notes:**

^a^. Data available for 68 of 72 younger patients and 48 of 56 older patients. The device does not provide an AAI when arrhythmia is detected during the recording or when pulse signal quality is insufficient.

Continuous variables are reported as median (IQR). P value was calculated using Mann–Whitney U test. AAI = autonomic arousal index; IQR = interquartile range.

The autonomic arousal index (AAI) is calculated automatically by the polygraphy device from the photoplethysmographic pulse wave signal. An autonomic arousal is defined as a pulse rate increase ≥ 20%, a pulse wave amplitude attenuation ≥ 40%, or a pulse wave amplitude attenuation ≥ 35% combined with a pulse rate increase ≥ 15% relative to baseline.(Sommermeyer, Zou et al. 2012) The AAI was lower in older than in younger patients. The AAI reflects autonomic activation and should not be interpreted as a surrogate of cortical arousals: in data from our previous validation study of the device in psychiatric inpatients,(Bailer, Stein et al. 2024) the AAI did not correlate with the arousal index obtained by polysomnography (n = 57; rs = 0.09; unpublished data). The lower AAI in older patients may therefore reflect an attenuation of autonomic and vascular reactivity rather than less sleep fragmentation: in a general population sample, the frequency of pulse wave amplitude drops decreased with age and was lower in individuals with hypertension, diabetes, or previous cardiovascular events.(Hirotsu, Betta et al. 2020)

## 1.4 Supplementary Table 4. Comparison of AUCs for AHI ≥ 15

|  | **AUC (95% CI)** | **AUC (95% CI)** | **Δ AUC** | **p** |
| --- | --- | --- | --- | --- |
|  | ***Younger Patients*** | ***Older Patients*** |  |  |
| STOP-Bang | 0.52  (0.28–0.76) | 0.56  (0.38–0.74) | -0.04 | .8 |
| STOP-B28 | 0.38  (0.13–0.63) | 0.61  (0.42–0.79) | -0.23 | .2 |
| BOAH | 0.63  (0.41–0.85) | 0.59  (0.43–0.75) | 0.04 | .8 |
| GOAL | 0.65  (0.47–0.82) | 0.46  (0.29–0.63) | 0.19 | .1 |
| NoSAS | 0.64  (0.43–0.85) | 0.47  (0.31–0.63) | 0.17 | .2 |
| No-Apnea | 0.61  (0.41–0.82) | 0.46  (0.29–0.63) | 0.15 | .3 |

**Notes:**

ΔAUC indicates difference between AUCs; AHI = apnea–hypopnea index; AUC = area under the curve.

## 1.5 Supplementary Table 5. Sensitivity and Specificity of Binary Questionnaire Items for Any OSA (AHI ≥ 5)

| **Item** | **Sensitivity, % (95% CI)** | **Specificity, % (95% CI)** | |
| --- | --- | --- | --- |
| Snoring | 73.3 (62.4-82.0) | | 20.8 (12.0-33.5) |
| Daytime tiredness | 37.3 (27.3-48.6) | | 49.1 (36.1-62.1) |
| Observed apneas | 28.0 (19.1-39.0) | | 81.1 (68.6-89.4) |
| Hypertension | 69.3 (58.2-78.6) | | 54.7 (41.5-67.3) |
| Male sex | 64.0 (52.7-73.9) | | 45.3 (32.7-58.5) |

**Notes:**

Sensitivity and specificity were calculated in the entire cohort (n=128; 75 patients with and 53 patients without OSA). 95% confidence intervals were calculated using the Wilson score method. AHI = apnea–hypopnea index; CI = confidence interval; OSA = obstructive sleep apnea.

## 1.6 Supplementary Figure 1. Receiver operating characteristic curves of the questionnaires for moderate and severe OSA.

**Supplementary Figure 1.** ROC analysis for the diagnosis of moderate to severe OSA based on AHI ≥ 15 events/h. Solid line: younger patients; dashed line: older patients.

# 2 References

Bailer, M., E. M. Stein, M. I. Sprügel, S. Mestermann, P. Spitzer, J. Utz, S. Zirlik, F. S. Fuchs and J. Kornhuber (2024). "Portable polygraphic device (Somnocheck micro CARDIO®) provides accurate diagnostic information in psychiatric patients at risk for obstructive sleep apnoea: an observational cohort study." BMC Psychiatry **24**(1): 607.

Hirotsu, C., M. Betta, G. Bernardi, P. Marques-Vidal, P. Vollenweider, G. Waeber, V. Pichot, F. Roche, F. Siclari, J. Haba-Rubio and R. Heinzer (2020). "Pulse wave amplitude drops during sleep: clinical significance and characteristics in a general population sample." Sleep **43**(7).

Sommermeyer, D., D. Zou, L. Grote and J. Hedner (2012). "Detection of sleep disordered breathing and its central/obstructive character using nasal cannula and finger pulse oximeter." J Clin Sleep Med **8**(5): 527-533.
